# The Role of Mitochondrial Dysfunction in Vascular Disease, Tumorigenesis, and Diabetes

**DOI:** 10.3389/fmolb.2021.671908

**Published:** 2021-05-07

**Authors:** Olga A. Zhunina, Nikita G. Yabbarov, Andrey V. Grechko, Antonina V. Starodubova, Ekaterina Ivanova, Nikita G. Nikiforov, Alexander N. Orekhov

**Affiliations:** ^1^Chemical Biology Department, Russian Research Center for Molecular Diagnostics and Therapy, Moscow, Russia; ^2^Federal Research and Clinical Center of Intensive Care Medicine and Rehabilitology, Moscow, Russia; ^3^Federal Research Centre for Nutrition, Biotechnology and Food Safety, Moscow, Russia; ^4^Department of Basic Research, Skolkovo Innovative Center, Institute for Atherosclerosis Research, Moscow, Russia; ^5^National Medical Research Center of Cardiology, Institute of Experimental Cardiology, Moscow, Russia; ^6^Institute of Gene Biology, Moscow, Russia; ^7^Laboratory of Cellular and Molecular Pathology of Cardiovascular System, Institute of Human Morphology, Moscow, Russia; ^8^Laboratory of Angiopathology, Institute of General Pathology and Pathophysiology, Moscow, Russia

**Keywords:** atherosclerosis, cancer, diabetes, DNA damage, mitochondria, reactive oxygen species, reactive nitrogen species

## Abstract

Mitochondrial dysfunction is known to be associated with a wide range of human pathologies, such as cancer, metabolic, and cardiovascular diseases. One of the possible ways of mitochondrial involvement in the cellular damage is excessive production of reactive oxygen and nitrogen species (ROS and RNS) that cannot be effectively neutralized by existing antioxidant systems. In mitochondria, ROS and RNS can contribute to protein and mitochondrial DNA (mtDNA) damage causing failure of enzymatic chains and mutations that can impair mitochondrial function. These processes further lead to abnormal cell signaling, premature cell senescence, initiation of inflammation, and apoptosis. Recent studies have identified numerous mtDNA mutations associated with different human pathologies. Some of them result in imbalanced oxidative phosphorylation, while others affect mitochondrial protein synthesis. In this review, we discuss the role of mtDNA mutations in cancer, diabetes, cardiovascular diseases, and atherosclerosis. We provide a list of currently described mtDNA mutations associated with each pathology and discuss the possible future perspective of the research.

## Introduction

During the last decades, it became evident that mitochondria have a broader biological significance than just energy production. The “powerhouse” of the cell is a semi-autonomous and dynamic organelle that possesses its own genome encoded by a circular mitochondrial DNA (mtDNA) that resembles a bacterial chromosome. According to current understanding, mitochondria probably derive from α-purple bacteria. The mitochondrial genome encodes four out of five enzyme complexes responsible for oxidative phosphorylation and energy production in the organelle (Frazier et al., 2019). Human mitochondrial genome is quite small, being 16569 base pairs long, and containing 37 genes that encode for 13 polypeptides, 2 ribosomal RNAs (rRNAs), and 22 transport RNAs (tRNAs) (Garcia et al., 2017; Li et al., 2019). Nuclear DNA encodes the other 79 polypeptides that are present in the mitochondria (Sharma et al., 2019). Mitochondrial dysfunction is observed in many human pathologies, and in most cases manifests itself in energy production (ATP synthesis) deficiency (Chen et al., 2019; Haas, 2019). Several mtDNA mutations leading to mitochondrial dysfunction have been identified to date. They occur both in regions encoding the respiratory chain enzymes and tRNAs and in non-coding regions, including the mtDNA D-loop, which is required for DNA polymerase binding and replication initiation (Li et al., 2019; Moro, 2019). Mutations in mtDNA may result in oxidative chain defects and subsequent pathology development. Mitochondria-related nuclear mutations also contribute to pathological processes, such as coenzyme Q (CoQ) deficiency, glutaric acidemia type 2, mtDNA depletion syndrome, mitochondrial neuro-gastrointestinal encephalopathy (MNGIE) and mitochondrial myopathy, encephalopathy, lactic acidosis, and stroke-like episodes (MELAS) (Patel et al., 2019; Yu et al., 2019).

Mutations in mtDNA have been reported in different human diseases affecting distinct organs and tissues, including malignancies, diabetes mellitus, and cardiovascular disorders (Bray and Ballinger, 2017; Hertweck and Dasgupta, 2017; Pinti et al., 2019). Establishing causal relationships between certain mtDNA mutations and pathological features is challenging because of the various roles and functions of the affected cell types. For instance, immune cells, platelets and endothelial cells are all involved in atherosclerosis progression (Bray and Ballinger, 2017). As an example, one of the widely used methods of mtDNA analysis, whole mtDNA genome amplification and subsequent evaluation of mtDNA integrity, does not allow assessing the possible causal link with pathological features (Duan et al., 2019).

Several chronic human pathologies were found to be associated with various mtDNA deletions and point mutations. Various types of cancer occupy a special place among them. Typically, malignant calls have altered metabolic profile, with increased rates of glycolysis due to high glucose consumption and decreased rates of oxidative phosphorylation. This persistence of aerobic glycolysis in the presence of oxygen is known as Warburg effect (Liberti and Locasale, 2016). The important role of the M2 splice isoform of pyruvate kinase in this effect has been reported (Locasale and Cantley, 2011). After the Warburg effect was discovered, its implication in ATP synthesis acceleration, tumor microenvironment and cell signaling has been hypothesized and proven. Mitochondrial DNA damage was found to contribute to this metabolic alteration through changes in mitochondrial redox potential, which increases reactive oxygen species (ROS) generation (Locasale, 2012; Kaplon et al., 2013).

**FIGURE 1:**
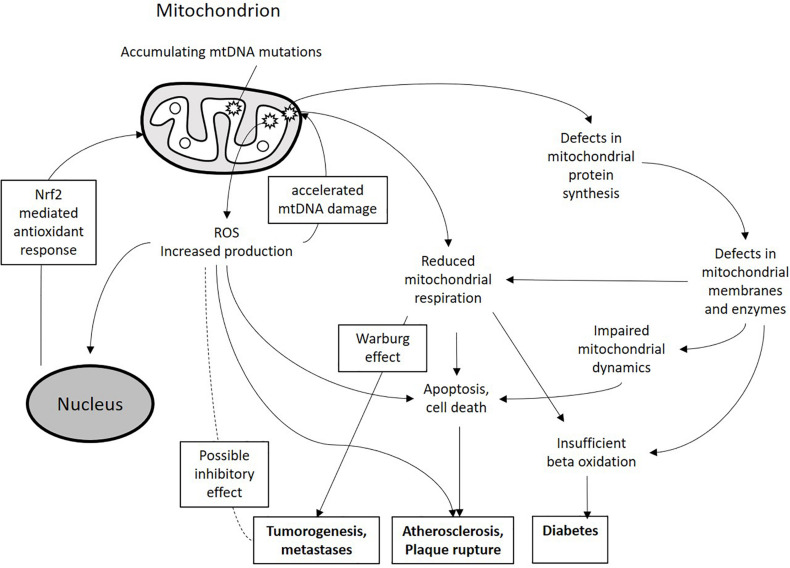
Schematic presentation of the miutohcondrial dysfunction involvement in chronic pathologies. Accumulating mtDNA mutations and associated defects in mitochondrial proteins can lead to increased ROS production, reduced mitochondrial respiration and impaired mitochondrial dynamics. These processes can lead to alteration of cellular metabolism and increased cell death and apoptosis thus contributing to the development of pathological phenotypes.

Mitochondria play a key role in normal and damaged cellular metabolism, energy production, cell signaling pathways, cell survival and apoptosis. Described mitochondrial functions are essential for all human organs and tissues in order to maintain homeostasis and oxygenation, which is especially important in mitochondrial cardiac pathology diagnosis and treatment (Martínez et al., 2017). Currently, mtDNA mutations are regarded as risk factors in different cancers, diabetes, coronary artery disease, vascular pathologies, cerebrovascular disease, and myocardial infarction (Jia et al., 2013; Szabó, 2013; Sinyov et al., 2017). These mutations may serve as important disease modifiers and therefore can potentially be used as therapeutic targets. Moreover, mtDNA mutations can be used for diagnostic purposes. With the rapid improvement of molecular genetic methods and cytoplasmic hybrid (cybrid) technology, studying of mtDNA mutations became easier and faster, while the development of mitochondria-targeting therapeutic approaches, such as mitochondrial antioxidants, opened new opportunities for correcting the associated mitochondrial defects. Therefore, clinical significance of mtDNA mutations is constantly growing. In this review, we attempt to summarize the current knowledge on mtDNA mutations in such chronic human diseases as cancer, diabetes, cardiovascular diseases and atherosclerosis (Figure 1).

## MtDNA Mutagenesis and Mitochondrial Dysfunction

In comparison to genomic DNA, mtDNA is less protected from damage and mutagenesis, and is especially susceptible to oxidative damage (Kaarniranta et al., 2019; Peoples et al., 2019). Unlike genomic DNA, which is protected and stabilized by histones, mtDNA lacks histone packaging. That may partially explain the increased susceptibility of mtDNA to mutagenesis, although this hypothesis remains controversial. For instance, mtDNA was found to be associated with transcription and packaging factor (TFAM) protein, which is important for mtDNA replication, but may play a stabilizing and protective role (Alam et al., 2003). A more likely source of increased mtDNA mutagenesis is its replication and repair mechanisms. An mtDNA replisome consists of DNA polymerase γ (Polγ), a helicase protein Twinkle, a single-stranded binding protein (mSSB) that covers the single strand during replication, and a number of additional proteins, including TFAM, transcription elongation factor (TEFM), exonuclease MGME1, DNA ligase III, mitochondrial RNA polymerase, and RNAse H1. The proposed mtDNA replication models provide some explanation for relatively high level of mutagenesis, including deletion and point mutation formation (Fontana and Gahlon, 2020). Since mtDNA is present in many copies and is replicated more frequently than genomic DNA, replication errors occur more often. In addition, Polγ is known by its relatively higher error rate. Sporadic mtDNA mutations can be subject to clonal expansion that had been demonstrated in some human tissues (Lawless et al., 2020). Possible explanations of increased mtDNA mutagenesis and accumulation of mtDNA mutations are described in several recent reviews (Fontana and Gahlon, 2020; Lawless et al., 2020). Although DNA repair mechanisms have been identified and partially characterized in mitochondria, their efficacy is lower than that of the nuclear DNA repair systems, which contributes to the higher rate of mutagenesis (Alencar et al., 2019).

The mtDNA mutation types include point mutations, deletions, and insertions. Changes in mtDNA copy number are also observed. Mutated mtDNA can be inherited by the daughter cells along with the normal mtDNA. In some situations, whole populations of cells may content only the mutated form of mtDNA, as can observed in some tumors (Srinivas et al., 2019). Mutation profile of mtDNA can vary not only in different tissues and organs of the individual, but also within the same organelle, depending on the amount of the mutant copies among the total pool of circular mtDNA molecules (heteroplasmy level). Heteroplasmy is explained by the large number of mitochondria per cell, that can each contain from 2 to 10 mtDNA copies, and can vary from 0 to 100%. Heteroplasmy level for a given mutation may not necessarily be the same in all tissues (Stefano and Kream, 2016; Burgstaller et al., 2018; Zhang et al., 2019). Interestingly, it was found that about 10% of wild type mtDNA was sufficient to support normal respiratory metabolism in MELAS (associated with m.3243A > G mutation). It has also been confirmed that in the case of tRNA mutations or deletions, 10% of wild type mtDNA was sufficient to sustain normal mitochondrial activity (Chomyn et al., 1991; Hayashi et al., 1991). Therefore, the threshold of heteroplasmy level for these mutations can be estimated to be about 90%. Mutation frequency in cases described beyond this threshold leads to various disorders. However, within this limit, respiratory chain activity and ATP synthesis can be maintained at a normal level. By contrast, some mtDNA mutations are characterized by low threshold levels of heteroplasmy, being more challenging to study (Gardner et al., 2015).

**FIGURE 2:**
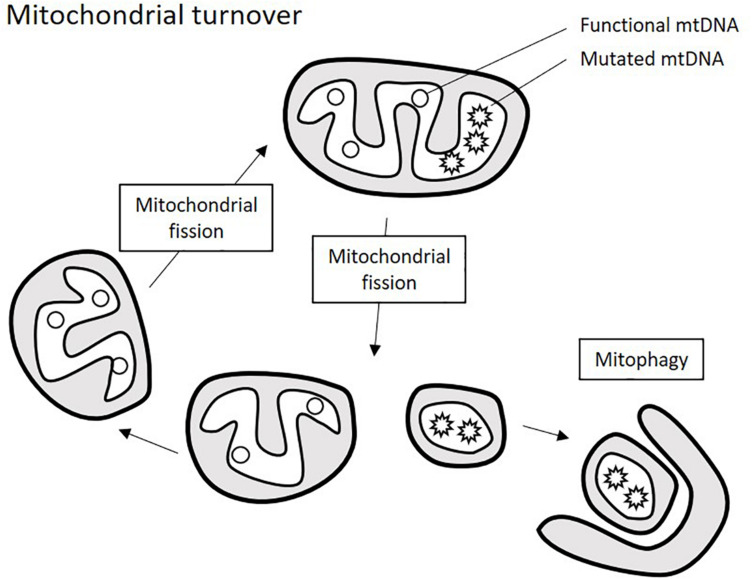
Schematic presentation of mitochondrial turnover. Mitochondria ungergo cycles of fission and fusion events that allow renewing mitochondrial population. Excessive or defective parts of the mitochondria are degraded through mitophagy, which requires prior fragmentation of them by mitochondrial fission.

The renewal of mitochondrial population within the cell is achieved by the finely balanced processes of mitochondrial turnover: fission, fusion, and mitophagy that are orchestrated by the key proteins: non-muscle myosin family II proteins (NMII), mitochondrial fission-1 protein (FIS1), mitochondrial fission factor (MFF), dynamin-1-like protein (DRP1), dynamin 2 (DNM2), mitochondrial dynamics protein 49 and 51 (MID49/51) transmembrane protein 135 (TMEM135), mitofusins 1 and 2 (MFN1/2), optic atrophy 1 (OPA1), and misato protein (MSTO1). Excessive or dysfunctional organelles are subject to fission into small fragments that can be destined for degradation through mitophagy (a specialized type of autophagy). Fragments that retain functionality can form renewed organelles through mitochondrial fusion (Figure 2). This turnover not only controls the number of mitochondria per cell, but also helps maintaining a functional pool of mtDNA, and imbalance of mitochondrial turnover is tightly linked to the increase of heteroplasmy for many known mtDNA mutations (Elorza and Soffia, 2021).

The role of ROS in mtDNA damage and, as a consequence, in increased mtDNA mutation rate had been proposed by early studies. However, solid evidence for such role is still lacking, and the significance of this process remains a matter of debate (Lawless et al., 2020). In favor of possible deteriorating effect of ROS on mitochondrial genome is the fact that inside the mitochondria, ROS are produced in physical proximity to mtDNA on the inner mitochondrial membrane by the respiratory chain enzymes that release them as by-products (Pichaud et al., 2019). Recent studies have shown that oxidative damage can directly affect mitochondrial Polγ, reducing its replication fidelity and, as a result, increasing the mtDNA mutagenesis rate by as much as 20-fold (Anderson et al., 2020). It is currently widely known that increased ROS production is a hallmark of mitochondrial dysfunction, which can be caused by mtDNA damage. Therefore, a vicious cycle can exist that drives pathological processes associated with mitochondrial dysfunction (Figure 1). It is likely that some organs and tissues that are highly dependent on mitochondrial energy production, including cardiac tissue and brain, are especially vulnerable to this process.

A special role of increased mitochondrial ROS production and oxidative damage was described for aging and related processes. It is currently well-recognized that oxidative stress plays a major role in aging processes and pathogenesis of age-related disorders. Telomeres shortening, one of the hallmarks of aging, can be accelerated by oxidative stress conditions and slowed down by antioxidants (Zia et al., 2021). Since mitochondrial dysfunction appears to a major source of oxidative stress in aging organs and tissues, it is naturally regarded as a promising therapeutic target to alleviate these processes. The use of mitochondrial antioxidant can make antioxidant therapy more selective. Another approach is stimulation of mitochondrial renewal and clearance of proteins, nucleic acids and whole organelles subjected to oxidative damage through enhanced mitophagy. Mitochondrial dysfunction-mediated aging appears to be especially relevant in organs and tissues with high energy demand, such as cardiac tissue (Elorza and Soffia, 2021; Zia et al., 2021). However, same processes are relevant for many other chronic human diseases, including cancer and diabetes.

## The Role of mtDNA Mutations in Cancer

### Metabolic Changes in Malignant Cells

Numerous studies have revealed associations between mtDNA mutations and different human malignancies (Porporato et al., 2014; Van Gisbergen et al., 2015). Malignant transformation of cells is accompanied by changes in energy metabolism, which depends on changes in mitochondrial function. The classical study of Otto Warburg has shown that cancer cells actively metabolize glucose and produce an excess of lactate even in the presence of oxygen. It was concluded that malignant cells have increased glycolysis rate that helps compensate defects in the mitochondrial respiratory chain (Liberti and Locasale, 2016). Early studies have described the role of Warburg effect in malignant cell proliferation, however, it was substantially revised in light of the more recent studies. It was found that a large amount of acetyl-CoA (Ac-CoA) entering the tricarboxylic acid cycle (TCA) is more likely to come from fatty acid oxidation (FAO), amino acid replenishment and gluconeogenesis pathway (Corbet and Feron, 2017). Thus, the main role of TCA shifts from supplying H + and redox equivalents to the downstream electron transport chain, but providing tumor cells with glutamine, proline, ornithine, lysine, methionine, and other non-essential amino acids required for biosynthesis (Kalhan, 2016; McDonnell et al., 2016). Moreover, recent studies have revealed that glutamine plays a crucial role in redox equivalent replenishment. However, the role that essential and non-essential amino acids in redox replenishment remains to be further elucidated (Metallo and Heiden, 2013; Fan et al., 2014). Mitochondrial oxidative phosphorylation directly regulates the malignant cell proliferation and metastases formation. It was suggested that ROS generated by the mitochondria sensitize the cells to anoikis therefore inhibiting metastases and affecting cell survival (Lu et al., 2015). Therefore, inhibition of glycolysis would favor the oxidative phosphorylation providing for anti-metastatic effect. The potential anti-tumor effect of oxidative stress is confirmed by the notion that nuclear factor-erythroid 2-related factor 2 (NRF2), a prominent transcriptional inducer of cellular antioxidant response, was shown to be protective for cancer cells through modulation of apoptosis and increased chemoresistance (Kahroba et al., 2019). Mitochondrial dysfunction caused by mtDNA mutagenesis and damage can also enhance tumorigenic potential of cells. For instance, it was shown that accumulation of mildly deleterious mtDNA mutations increased mitochondrial mass in Hodgkin lymphoma cell lines, apparently through a compensatory mechanism, and increase of tumor growth in mouse model (Haumann et al., 2020). Another recent study demonstrated that accumulation of mtDNA mutations and associated dysfunction of the mitochondrial respiratory chain contributed to the metabolic switch of cancer cells to aerobic glycolysis in fumarate hydratase-deficient renal cancer (Crooks et al., 2021). That was in line with the earlier observation that artificial depletion of mtDNA in colorectal cancer cells promoted aerobic glycolysis activation (Mou et al., 2018). Therefore, mitochondrial dysfunction caused by mtDNA damage results in a metabolic shift typical for many cancers, with increased glucose uptake and promotes tumor growth. Together, these observations confirm the important role of mitochondrial function in tumor cell metabolism and growth and indicate the relevance of mtDNA mutations for the development of future therapies.

### Germline mtDNA Mutations Associated With Cancer

Cancer-associated mtDNA mutations can occur both in the germline cells (germline mutations) and in somatic cells (Gammage and Frezza, 2019). Associations between germline mtDNA mutations and malignancies have been established by numerous studies. Some of these mutations were observed in specific type of cancer within certain populations. Human populations can bear different haplogroups of mtDNA with distinctive polymorphisms accumulated along the maternal line of inheritance (Torroni et al., 1996). Variations of mtDNA in a particular geographic area may result from genetic drift and adaptive selection (Mishmar et al., 2003). One study has classified 30 different haplogroups and demonstrated that the risk in the haplogroup of the M7b2 population was associated with increased risk of hematopoietic cancer (Verma et al., 2007). Another study analyzed the potential value of mtDNA variations as markers of bladder cancer (Cormio et al., 2017). In this work, some germline mtDNA mutants found in the blood were used as markers for predicting the risk of bladder cancer development. The wide range of mtDNA mutations and tumor-specific mtDNA variants appear to be promising for the search of new molecular markers for the detection of bladder cancer at the early stages. Analysis of mtDNA in blood samples and neoplastic tissues of patients with bladder cancer showed that variant m.16069C > T in the D-loop was associated with high risk of disease development (Shakhssalim et al., 2013). Petros with co-authors demonstrated associations of four different germline mutations in subunit I of cytochrome oxidase (m.6253T > C, m.6340C > T, m.6261G > A, and m.6663A > G) with prostate cancer (Petros et al., 2005). Lam with co-authors detected the association of variant m.5460G > A encoding the non-synonymous substitution of A331 to T in the ND2 protein with pancreatic cancer (Lam et al., 2012).

### Somatic mtDNA Mutations Associated With Cancer

A number of studies has reported mtDNA somatic mutations associated with hematological tumors, such as myelodysplastic syndromes, chronic lymphocytic leukemia, chronic myeloid leukemia (CML), acute myeloid leukemia (AML), and acute lymphoblastic leukemia (ALL) (Ju et al., 2014). Next generation sequencing approach proved to be useful for identifying malignancy-associated mtDNA mutations in patients with CML (Wulfert et al., 2008). Another study identified somatic mtDNA mutations in patients with lung cancer using paired comparative analysis of sequences of normal and tumor genomes obtained using Affymetrix Mitochondrial Resequencing Array 2.052. The study identified 532 somatic mutations distributed at 499 positions with very low overall frequency (1.07/bp). However, non-synonymous mutations leading to amino acid substitution were more frequent (1.83/bp), especially at two positions: 8701 (in the ATPase6 gene) and 10398 (in the NADH dehydrogenase 3 gene) (10.5/bp). Together, these mutations were observed in 86% of disease cases. The connection between the two most common mutations may indicate their simultaneous selection, perhaps because of their role in cancer development (Pagani et al., 2017). At the same time, the association of m.10398G > A mutation with cancer appears to vary across populations. A case-control study and meta-analysis conducted in Southern India did not reveal association of this mutation with breast cancer risk (Francis et al., 2013), while an earlier study confirmed the association of this mutation with breast and esophageal cancer risk in another population (Darvishi et al., 2007).

Another study analyzed ND4 mutation in transitional cell carcinomas (TCCs) of the upper urinary tract and normal perirenal soft tissues using DNA sequencing, restriction fragment length polymorphism (RFLP) analysis and denaturing high-performance liquid chromatography (DHPLC) (Tzen et al., 2007). The study detected ND4 mutations in 24.5% of tumors, while 11 of these mutations were also detectable in normal tissues by DHPLC, suggesting that tumor-associated mtDNA mutations can be present in normal tissues at low levels that are not detected by less sensitive methods such as DNA sequencing.

The study of hepatocellular carcinomas (HCCs) has described 13 somatic mtDNA mutations in 11 HCC samples. Of these mutations, 6 (m.6787T > C, m.7976A > G, m.9263A > G, m.9267G > A, m.9545A > G, and m.11708A > G) were homoplasmic, while 7 (956delC, m.1659T > C, m.3842G > A, m.5650G > A, 11032delA, 12418insA, and a 66 bp deletion)—heteroplasmic. The 11032delA and 12418insA mutations can cause frame-shifting in the ND4 and ND5 genes. The T1659C transition in the tRNAVal gene and m.5650G > A in the tRNA-Ala gene are known to be associated with some mitochondrial disorders. Mutations m.6787T > C (cytochrome c oxidase subunit I, COI), m.7976G > A (COII), m.9267G > A (COIII), and m.11708A > G (ND4) cause amino acid substitutions in highly conservative regions of the corresponding genes. In total, 76.9% of mtDNA mutations may cause mitochondrial dysfunction in HCCs. The authors suggested that mtDNA mutations can be present at a higher frequency in HCC than in normal tissues of the same individuals (Yin et al., 2010).

### The Role of mtDNA Mutations in Diabetes

Mitochondrial diabetes (MD) is a group of genetically determined diseases inherited through the maternal line, that are manifested by a combination of hyperglycemia and associated neurological diseases, such as hearing loss, myopathy, and neurologic disorders (Karaa and Goldstein, 2015). The role of mitochondria and mtDNA mutations as causative agents of diabetes has been extensively studied (Crispim et al., 2008; Tabebi et al., 2016). It was shown that MD can be caused by point mutations, deletions and duplications in mtDNA that affect functioning of certain genes (Brandon et al., 2005; Crispim et al., 2008; Tabebi et al., 2016). Although nuclear DNA mutations also play an important role in the pathology development, they appear with a 10–1,000 times lower frequency than mutations in mtDNA (Guillausseau et al., 2001; Katulanda et al., 2008).

The most common MD-associated mutation is m.3243A > G in the mitochondrial tRNA-Leu gene MT-TL1, which affects the overall level of mitochondrial oxidative phosphorylation (McMillan et al., 2019). This mutation was found to be associated with maternally inherited diabetes and deafness (MIDD), a rare form of diabetes with a prevalence varying from 0.5 to 3% among different ethnic groups (Crispim et al., 2008). MIDD patients usually present with impaired hearing, cardiomyopathy, neurological abnormalities and renal insufficiency. Early detection of the disease-causing mutation is important for providing appropriate clinical care for these patients. Detection of m.3243A > G mutation in blood and urine sediments from MD patients can be performed based on pyrosequencing. A study conducted in Chinese Han patients demonstrated that m.3243A > G mutation was carried by 1.69% of diabetic patients, but was not detected in healthy control persons enrolled in the study (Wang et al., 2013). Moreover, in the diabetes patient group from a different population, two double mutations were identified: m.3243A > G + m.3394T > C and m.3243A > G + m.12026A > G (El-Hattab et al., 2012a, 2014). Study of molecular mechanisms of DM revealed that m.3243A > G mutation may play a role in altering glucose metabolism. Stable isotope study of diabetic and non-diabetic individuals carrying m.3243A > G mutation showed that probable cause of MD is the combination of insulin resistance and relative insulin deficiency in individuals carrying the m.3243A > G mutation (El-Hattab et al., 2012b).

MELAS syndrome (mitochondrial encephalopathy with lactic acidosis and stroke-like episodes) is a genetic disease with damage to the central nervous system, muscle tissue and various organs. The pathology is based the impairment of tissue respiration and deficient energy metabolism. The clinical picture is heterogeneous, and includes acute episodes resembling a stroke, epileptic seizures, exercise intolerance due to muscle weakness. MELAS syndrome refers to diseases caused by mtDNA defects that lead to deficient energy production in the mitochondrial respiratory chain. According to various sources, the pathology occurs with a frequency of 1:15,000 to 1:20,000 people, affecting both sexes and manifesting itself at the average age is 6–10 years. To date, about 10 genes which mutations are associated with MELAS are known. Among them, mutations in genes encoding tRNA are most frequently detected. In most cases (80–90%), it is the m.3243A > G mutation (El-Hattab et al., 2012a, b, 2014). This mutation appears to be quite common across human populations, and is associated with different pathological phenotypes, including, apart from MELAS and MIDD, myopathy. Recent studies provided some insight on metabolic changes caused by this mutation revealing common metabolic features of MELAS and MIDD, but also important differences between these phenotypes. While MIDD is characterized by altered glucose metabolism, which is not the case in MELAS, the latter is associated with altered fatty acid oxidation (Esterhuizen et al., 2021). The reasons for such differences are not yet fully understood. Due to the heteroplasmy effect, presence of a mutation does not necessarily lead to the phenotypic manifestation of the disease. A large number of defective mtDNA copies increases the likelihood of clinical manifestation of the syndrome. According to the severity of clinical manifestations, three forms of MELAS syndrome are distinguished: asymptomatic carriage (presence of a genetic mutation and changes during muscle biopsy against the complete absence of clinical signs of the disease), oligosymptomatic (individual components of the syndrome are detected, such as muscle weakness or headaches), manifest is a vivid clinical picture with acute episodes (El-Hattab et al., 2015).

A number of other mitochondrial mutations have been reported to be associated with diabetes mellitus. A recent study has identified m.4216T > C and m.5178C > A mutations (that affect respectively the *ND1* and *ND2* genes) in maternally transmitted diabetes (Jiang et al., 2021). These mutations were found to cause mitochondrial dysfunction and oxidative stress contributing to the disease pathology. It is currently well-recognized that oxidative stress affects pancreatic β-cells, being one of the drivers of diabetes development. Increase of ROS production linked to mitochondrial dysfunction in this cell type was recently recognized as a one of the pathological effects of streptozotocin, which is used for artificial diabetes induction in mouse models (Al Nahdi et al., 2017). It is therefore possible that future studies will lengthen the list of mtDNA mutations associated with diabetes mellitus.

## MtDNA Mutations Associated With Neurodegenerative and Cardiovascular Diseases

Deficiency of mitochondrial energy production associated with mtDNA mutations is especially impactful in organs and tissues characterized by naturally high energy consumption, such as muscle and neuronal tissues. In these tissues, mtDNA mutations can lead to myopathy, atrophy and progressive degeneration processes. The first described human disease caused by mtDNA missense mutation was Leber’s hereditary optic neuropathy (LHON). Later studies showed that several missense mtDNA mutations were associated with the syndrome. However, the most common remains the m.11778G > A mutation that affects NADH dehydrogenase (complex I, subunit 4) changing a highly conserved arginine to histidine (Cruz-Bermúdez et al., 2016). Another example is the m.8993T > G mutation associated with maternally inherited Leigh syndrome (subacute necrotizing encephalopathy) and NARP (neuropathy, ataxia, and pigmentary retinopathy) that affects the ATPase6 subunit (complex V) by changing a hydrophobic leucine to an arginine. Interestingly that Leigh syndrome developed only in individuals carrying the mutation at heteroplasmic level above 90%. At the same time, individuals with heteroplasmy level between 70 and 90% mainly developed a less severe NARP syndrome. Individuals harboring heteroplasmic levels below 70% did not develop any Leigh or NARP syndrome symptoms (Weerasinghe et al., 2018).

Cardiomyopathy and related disorders are commonly caused by mtDNA mutations interfering with mitochondrial protein synthesis (tRNA, rRNA). These mutations are typically associated with multisystemic disorders. The most severe syndromes are usually represented by mitochondrial myopathy and myoclonic epilepsy with ragged red fibers (MERRF syndrome). Known mutations affecting mitochondrial protein synthesis include transitions m.8344A > G in the tRNA-Lys gene and m.3243A > G in the tRNA-Leu gene. Both are associated with MERRF and MELAS syndromes (Chou et al., 2018). Patients with MERRF syndrome typically present with cardiomyopathy, dysrhythmia and neuropathy. About 80% of MERRF patients harbor the m.8344A > G mutation. The remaining 20% carry m.8356T > C and m.8363G > A tRNA-Lys mutations. Some well-characterized mutations associated with maternally transmitted mitochondrial diseases are summarized in Table 1.

**TABLE 1:** Some common mtDNA mutations associated with hereditary mitochondrial diseases.

**Mutation(s)**	**Location**	**Disease**	**References**
m.3243A > G	tRNA–Leu gene (UUR recognition codon)	Maternally transmitted diabetes, mitochondrial encephalopathy, lactic acidosis, and stroke-like episodes (MELAS), myopathy	Crispim et al., 2008
m.4216T > C m.5178CA	ND1 ND2	Maternally transmitted diabetes	Al Nahdi et al., 2017
m.11778G > A	NADH dehydrogenase	Leber’s hereditary optic neuropathy	Cruz-Bermúdez et al., 2016
m.8993T > G	ATPase6 subunit	Maternally inherited Leigh syndrome (>90% heteroplasmy level), neuropathy, ataxia, and retinitis pigmentosa (NARP) syndrome (70–90% heteroplasmy level)	Weerasinghe et al., 2018
m.8344A > G m.8356T > C m.8363G > A	tRNA-Lys gene	Myoclonic epilepsy with ragged red fibers (MERRF)	Chou et al., 2018

Apart from point mutations, rearrangement of mtDNA can also cause a variety of disorders including heart block, seizures and lethal pediatric pancytopenia. It was shown that mtDNA deletions accumulate throughout life, especially in damaged tissues. Several studies revealed that mtDNA rearrangements, deletions or copy number changes may be influenced pharmaceutically. As an example, doxorubicin and zidovudine possess notorious cardiotoxicity that can be partially attributed to that effect (Varga et al., 2015; Ferdinandy et al., 2019).

Age-related changes in oxidative phosphorylation and somatic mtDNA mutations accumulate gradually through life. These changes are partially explained by ROS and RNS-mediated damage that also affects proteins and membranes interfering with complex structural integrity and contributing to cellular dysfunction and disease development. Two protein players are known to directly affect cellular and mitochondrial antioxidant system are NRF2 (mentioned above) and Uncoupling protein 2 (UCP2), which is a mitochondrial anion carrier expressing in most tissues. UCP2 uncouples oxygen consumption from ATP synthesis. Besides antioxidant function, UCP2 participate in DRP1-dependent mitochondrial fission. Recent studies revealed UCP2 and Nrf2 deficiency involvement in impaired mitochondria function associated with cardiovascular diseases (Holmström et al., 2016; Tian et al., 2018).

## The Role of mtDNA Mutations in Atherosclerosis

Atherosclerosis is a chronic inflammatory disease characterized by lipid accumulation and local inflammatory process in the arterial wall leading to vessel occlusion and thrombosis (Fava and Montagnana, 2018). Mitochondrial involvement in atherosclerosis pathogenesis has been studied by several groups (Yu and Bennett, 2016; Docherty et al., 2018; Peng et al., 2019). The mechanisms of such involvement include impairment of the electron transport chain and ROS degradation systems, increased apoptosis, unstable plaque formation and increased risk of thrombosis.

It has been clearly shown that elevated ROS level correlated with different stages of atherosclerosis leading to protein, lipids and mtDNA modification. In turn, mtDNA mutational status correlated with plaque formation (Yu et al., 2013; Sinyov et al., 2017). However, mtDNA damage also had a direct effect, independent of ROS generation, on atherosclerosis development (Yu et al., 2013).

The causative role of ROS-mediated damage in atherosclerosis development has been demonstrated in studies using apolipoprotein E knock-out (*apoE*^–^/*^–^*) mice (Di Marco et al., 2014). *ApoE* knockout mice present with fatty streaks and spontaneously developing fibrous plaque-like lesions similar to human atherosclerotic lesions. One of the useful models of atherosclerosis development is the mice line carrying mutated mitochondrial DNA polymerase with impaired activity, which leads to extensive and unspecific accumulation of mtDNA mutations. Studies of these models revealed the contribution of elevated ROS levels and mtDNA damage to the progression of atherosclerotic symptoms. Mitochondrial DNA damage was shown to be an early event in the vascular wall of *apoE*^–^/*^–^* mice, and was also present in circulating cells. Accumulating deletions and mutations of mtDNA were detectable long before the clinical symptoms of atherosclerosis became evident. It was found to play a pro-atherogenic role independently from ROS (Yu et al., 2013; Tian et al., 2016).

In humans, mtDNA mutations associated with atherosclerosis were studied in post-mortem tissues of atherosclerotic lesions in the smooth muscle cells. One study has revealed a deletion at 4977 position and missense mutations m.1555A > G, m.3256C > T, m.3336T > C, m.13513G > A, m.15059G > A, m.12315G > A, m.14459G > A, and m.5178C > A. Noteworthy, in contrast with previous studies, mutations m.1555A > G and m.14846G > A negatively correlated with atherosclerotic degeneration (Volobueva et al., 2019). A study conducted on patient blood samples revealed the presence of m.9477G > A, m.3243A > G and m.3256C > T mutations in leucocytes that were also observed in plaque tissues and correlated with atherosclerosis. Mutations m.3336T > C, m.12315G > A, m.13513G > A, m.14459G > A, m.14846G > A, and m.15059G > A correlated with the size of atherosclerosis plaques. Noteworthy, the heteroplasmy level of several of these mutations correlated with the patient age (Volobueva et al., 2019).

Several studies revealed that malfunction in dynamic mitochondria fusion and fission processes may be associated with elevated ROS production. Oxidative stress stimulates mitochondrial fusion. Mitochondrial fission was shown to play an important role in macrophage-mediated clearance of apoptotic cells (efferocytosis), deficiency of which may be responsible for pathological processes taking place in advanced atherosclerotic plaques (Wang et al., 2017). A very recent study has shown that coronary artery disease patients revealed in-creased levels of fusion markers MFN1, MFN2 and OPA1. Noteworthy, simultaneously increased levels of fission markers p-DRP1 and t-DRP1 were observed. The authors suggested that the observed changes indicated a shift from oxidative phosphorylation to-wards increased glycolysis in patients’ mitochondria (Ait-Aissa et al., 2019).

Increase of mtDNA damage reduces steady-state mitochondrial mRNA transcripts, protein synthesis rate and quality, influences the membrane potential and decreases total ATP pool in the endothelial and vascular smooth muscle cells cultures exposed to ROS (Mikhed et al., 2015). It was shown that 4-hydroxynonenal, a product of membrane lipid peroxidation involved in atherosclerosis progression, induced cell apoptosis via mitochondrial malfunction and elevated ROS production (Zhong and Yin, 2015). By contrast, elevated ROS generation was a characteristic of haploinsufficiency of superoxide dismutase 2 isoform and reduced aconitase activity in both basal and agonist-stimulated conditions enhancing vascular smooth muscle cells proliferation (Wang et al., 2012).

Together, these findings confirm the important causative role of mitochondrial dysfunction in atherosclerosis development and indicate that antioxidant agents may prove to be useful as preventive agents. Although clinical studies failed to demonstrate the benefits of commonly used antioxidants for patients with developed atherosclerosis, more research is needed to better understand the mechanisms of oxidative damage in atherosclerosis and, probably, develop targeted antioxidant therapies that may help at the beginning stages of the disease (Toledo-Ibelles and Mas-Oliva, 2018).

## Future Perspectives

Studying of mitochondrial dysfunction and mtDNA mutations in various human diseases is a rapidly evolving research field. The list of mtDNA mutations associated with one or another pathology is constantly growing. The relatively recently developed cybrid technology allows evaluating the impact of newly identified mtDNA variants impact on the development of mitochondrial dysfunction. Cybrids are created by fusion of enucleated cytoplast containing mitochondria carrying mtDNA variants of interest with cells depleted of mtDNA by treatment with mtDNA replication inhibitors (such as ethidium bromide) followed by a selection step. Such models allow studying the effects of mtDNA mutations in a constant nuclear genetic background (Wilkins et al., 2014). The cybrid models of Parkinson’s disease, MELAS, LHON, Alzheimer’s disease, Leigh syndrome and atherosclerosis have been already developed and characterized (Arduíno et al., 2015; Jeong et al., 2015; Jiang et al., 2016; Sazonova et al., 2019). The importance of cybrid models is highlighted by the great phenotypic heterogeneity associated with mtDNA mutations described in these pathologies. The reasons for such heterogeneity are often insufficiently understood, and the relative input of the mtDNA variant of interest and nuclear genetic background are sometimes difficult to distinguish. Another powerful tool for studying individual mtDNA variants is the recently developed single-cell mtDNA genotyping approach (Lareau et al., 2020). This high-throughput method allows distinguishing mtDNA heteroplasmy in individual cells and studying the presence of mtDNA mutations in multiple single cells, as well as their clonal tracing.

Methods of mtDNA damage correction are also being developed. Stimulation of mitochondrial population renewal through mitochondrial turnover and mitophagy is one of them, but more targeted approaches have appeared recently. Mitochondria-targeted transcription activator-like effector nucleases (mito-TALENs) can help reducing the mutational load for a certain mtDNA mutation in a similar way as previously developed TALENs for nuclear gene editing. As in most mitochondria-targeting therapies, selective delivery of the agent to the organelle was one of the challenges of this approach. A successful recent example of mito-TALENs technology was based on using AAV9 vectors for the delivery of mito-TALENs in mice. Using this approach, the authors succeeded in correcting of tRNA-Ala deficiency caused by the m.5024C > T mutation (Bacman et al., 2018).

It is currently well-understood that disturbances of oxidative phosphorylation and imbalanced ROS production play a central role in most of the conditions associated with mitochondrial damage and therefore appear to be promising points of therapeutic intervention. While general antioxidants were shown to be inefficient in many cases (Oyewole and Birch-Machin, 2015), specific mitochondria-targeted antioxidants revealed great potential for mitochondrial ROS elimination (Broome et al., 2018). The therapeutic potential of these agents needs to be explored by the future studies.

## Conclusion

Mitochondrial dysfunction is currently recognized as an important causative agent in the development of numerous chronic human pathologies. Improvement of DNA sequencing techniques and establishment of cybrid creation techniques broadened our knowledge about the specific mechanisms and mtDNA mutations involved in the pathologies. To date, several mtDNA mutations strongly associated with different cancers, diabetes and atherosclerosis have been described and characterized. Many of these mutations are linked to the excessive generation of ROS and oxidative stress, while others result in deficiencies of protein synthesis in the mitochondria resulting in metabolic and structural changes in the affected organelles. Future research should focus on evaluating the diagnostic and therapeutic value of these mutations and related mitochondrial dysfunction and designing novel therapeutic approaches targeting mitochondria.

## Author Contributions

AO, OZ, AS, and NN: conceptualization. AO and AG: resources. OZ, NY, and EI: writing—original draft preparation. EI and AS: writing—review and editing. EI: visualization. AO and NN: supervision and funding acquisition. AO: project administration. All authors have read and agreed to the published version of the manuscript.

## Conflict of Interest

The authors declare that the research was conducted in the absence of any commercial or financial relationships that could be construed as a potential conflict of interest.
